# A case of *Candida auris* candidemia in Xiamen, China, and a comparative analysis of clinical isolates in China

**DOI:** 10.1080/21501203.2021.1994479

**Published:** 2021-10-31

**Authors:** Jian Bing, Sijia Wang, Heping Xu, Shuru Fan, Han Du, Clarissa J. Nobile, Guanghua Huang

**Affiliations:** a Department of Infectious Diseases, Huashan Hospital and State Key Laboratory of Genetic Engineering, School of Life Sciences, Fudan University, Shanghai, China; b Department of Clinical Laboratory, First Affiliated Hospital of Xiamen University, Xiamen, China; c Institutes of Biomedical Sciences, Fudan University, Shanghai, China; d Department of Molecular and Cell Biology, University of California, Merced, CA, USA; e Health Sciences Research Institute, University of California, Merced, CA, USA

**Keywords:** *Candida auris*, antifungal resistance, mating type locus, virulence, morphology

## Abstract

The recently emerged fungal pathogen *Candida auris* often displays resistance to one or more antifungal drugs. Its infections have been identified in at least 40 countries on six continents to date. Here we report a case of *C. auris* candidemia in a patient in Xiamen, a city in south China. We also review currently reported cases of *C. auris *infection in China and compare the genetic and biological features of *C. auris *strains isolated from this country. Our phylogenetic analysis indicates that there are at least two *C. auris *genetic clades present in China (the South African clade and the south Asian clade) that display opposite mating type loci (one is *MTL***a** and the other is *MTL*α). We also found that there are several distinct features among the clinical isolates studied, including the expression of virulence factors, antifungal susceptibilities, and cellular morphologies, and that these features could be associated with the mating-type of the isolate. For example, *C. auris* *MTL***a** isolates generally secreted higher levels of secreted aspartyl proteases (Saps) at ambient environmental temperatures. Taken together, this study demonstrates that *C. auris* clinical isolates from China exhibit diversity in both biological and genetic features.

## 1. Introduction

The “superbug” fungus *Candida auris* is becoming a serious global public health threat (Sardi et al. 2018; Du et al. 2020). It was first identified as a novel fungal species and reported in 2009 by a Japanese group (Satoh et al. 2018). Due to its multidrug resistance and rapid prevalence in clinical or healthcare settings, the CDC has issued a couple of alerts to inform the awareness of *C. auris* infection. In the past decade, great progresses have been made on the study of this species, yet many aspects of its biology, genetics, and epidemiology remain to be investigated. As of February 2021, *C. auris* infections have been identified in at least 40 countries on six continents. Whole-genome sequencing (WGS) analyses indicate that four major clades of *C. auris* have emerged independently in four distinct geographical regions (Africa, South America, east Asia, and south Asia) (Lockhart et al. 2017; Du et al. 2020; Ahmad and Alfouzan 2021). In addition, a potential new clade was recently isolated from Iran. Over 200,000 SNPs had been identified between the Iran clade and the other clades (Chow et al. 2019).

The first case of *C. auris* infection in China was reported in 2018 by us (Wang et al. 2018). Subsequently, seventeen additional cases have been reported in Beijing and Shenyang, China (Chen et al. 2018; Tian et al. 2018). Thus far, of the reported *C. auris* strains in mainland China, only a single strain (BJCA001) belongs to the South Asian clade, and the other strains belong to the South African clade. Interestingly, strain BJCA001 is susceptible to all antifungal drugs tested, while the other seventeen strains are resistant to fluconazole based on their minimum inhibitory concentration (MIC) breakpoints (Chen et al. 2018; Tian et al. 2018; Wang et al. 2018). Here we report the first case of *C. auris* candidemia from a patient in Xiamen, a city in south China. We also reviewed all reported cases of *C. auris* infection to date in mainland China and performed genetic and biological comparative analyses of all known *C. auris* clinical isolates in China.

## 2. Materials and methods

### 2.1 Culture conditions

*Candida auris* strains were routinely grown in YPD medium supplemented with 5 μg/mL of phloxine B. The mating-type loci (*MTL*) of all *C. auris* strains used in this study were determined by PCR chemotyping assays using primers MTL**a** (5ʹ-TGACTCTTGAACAGCTTACG-3ʹ and 5ʹ-ATCTCGCAATGACCTCGTAC) and MTLα: (5ʹ-ATGCGATGCTAGTATGGATG-3ʹ and 5ʹ-ATGGTGCTTCCTTTTCTGTG-3ʹ).

Minimal inhibitory concentration (MIC) assays were performed according to the CLSI M27 (third edition) and our previous publication (Bing et al. 2020).

### 2.2 Phylogenetic analyses

The internal transcribed spacer (ITS) ribosomal regions of *C. auris* strains were aligned using mafft v7.015b26 (Katoh and Standley 2013). The ITS sequences were amplified from *C. auris* genomic DNA using primers ITS-F 5ʹ-GTCGTAACAAGGTTTCCGTAGGTG-3ʹ and ITS-R 5ʹ-GGTCCGTGTTTCAAGACGG. As described previously (Wang et al. 2018), based on General Time Reversible (GTR) and Gamma distribution with Invariant sites (G + I) models, we generated the maximum-likelihood phylogenetic tree using the tool RAxML v7.3.227 (Stamatakis 2014). The ITS sequences of the previously reported representative strains were obtained from the GenBank database (https://www.ncbi.nlm.nih.gov/) based on previous publications (Lockhart et al. 2017; Chen et al. 2018; Tian et al. 2018; Wang et al. 2018; Chow et al. 2019).

### 2.3 Secreted aspartyl protease (Sap) activity assay

As described previously (Wang et al. 2018), Sap activity of *C. auris* strains was determined using YCP-BSA assays at 25°C or 37°C. Approximately 5 × 10^6^ cells of *C. auris* in 5 μL ddH_2_O were spotted and cultured on YCB-BSA medium plates for seven days. The width of the white halos (BSA precipitation), which reflect the activity of Saps, was measured.

## 3. Results and discussion

### 3.1. Case presentation

A 67‐year‐old man with a 10-year history of gastric ulcers, gastric mucosal haemorrhaging and diabetes was admitted to the First Affiliated Hospital of Xiamen University. He presented with abdominal pain and melaena and was faecal occult blood test positive (OB+). A routine blood culture examination and a computerised tomography (CT) scan were performed. His level of C-reactive protein (CRP) was 16.7 mg/L. He was then diagnosed with gastric mucosal haemorrhaging, peritonitis, and bronchitis. Haemostasis was achieved and piperacillin/tazobactam and fluconazole were given prophylactically to prevent bacterial and fungal infections, respectively. On day six of admission, the patient presented with serious abdominal pain and an elevated body temperature of 39.2°C. Increased redness and swelling around the abdominal drainage tube was observed. Fungal cultures indicated that both the blood and drainage tube tip were positive for yeast species. The isolates were identified as *C. auris* by MALDI-TOF MS (Girard et al. 2016) and verified by sequencing of the ITS ribosomal region. The ITS sequences of the *C. auris* isolates from the blood and drainage tube tip were identical, suggesting that they were likely derived from the same clonal strain. The *C. auris* strain isolated from the blood was called XM1805 and the *C. auris* strain isolated from the drainage tube tip was called XM1803. Both isolates were not susceptible to fluconazole (MIC ≥128 mg/L) but showed a low MIC to caspofungin (≤0.25 mg/L). MIC assays for additional antifungal drugs were also performed, and these results are presented in supplementary Table 1. Since the *C. auris* isolates identified were resistant to fluconazole, the administration of fluconazole was stopped and caspofungin (50 mg/day) was administered to the patient for fourteen days. On days ten and twenty-two of admission, blood culture test results were negative for fungi. On day 31, the patient was discharged from the hospital. We note that the patient had a recent travel history to Japan but had not travelled to Shenyang or Beijing, China, two cities where *C. auris* had been previously isolated.

### 3.2. *Phylogenic and MIC analyses of* C. auris *clinical isolates from China*

A total of twenty known clinical isolates from China, including fifteen from Shenyang, three from Beijing, and two from Xiamen (XM1803 and XM1805), were obtained. We performed comparative biological analyses of all isolates and found that there are several distinct features among the different strains, including the expression of virulence factors, antifungal susceptibilities, and cellular morphologies.

**Figure 1.**
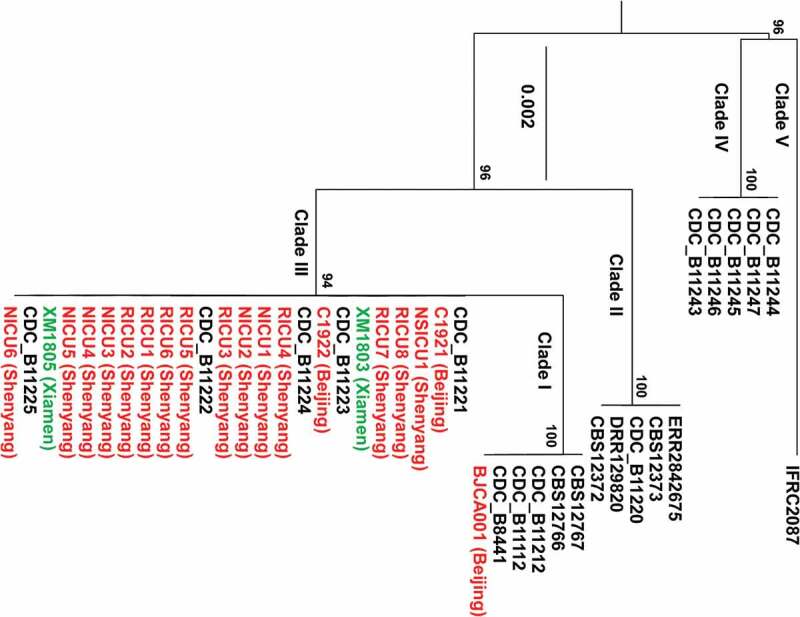
Phylogenic analyses of the *C. auris* strains isolated from China compared to other *C. auris* strains. The two strains (XM1803 and XM1805) isolated from Xiamen, China, are highlighted in green and the other isolates from China are highlighted in red. Twenty-one additional *C. auris* isolates representing the five genetic clades are also included (highlighted in black). The maximum-likelihood phylogenetic tree was generated based on ITS sequences. The scale bar indicates the number of nucleotide substitutions per site.

A summary of clinical information of the two *C. auris* isolates from Xiamen and the previously reported isolates from mainland China is presented in supplementary Table 2. Phylogenic analyses were performed based on ITS sequences of all *C. auris* isolates from China. Twenty-one *C. auris* strains from other countries representing the five reported genetic clades were also included in the phylogenic tree. As demonstrated in Figure 1, only strain BJCA001 from Beijing belongs to the South Asian clade, while the other nineteen isolates from China are closely related to the South African clade. This finding suggests that the South African clade is the predominant *C. auris* genetic clade in China.

**Figure 2.**
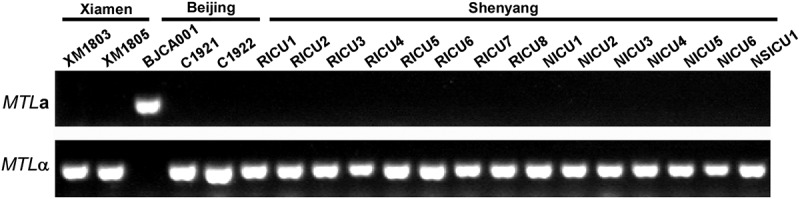
PCR analyses of the *MTL* loci of the different *C. auris* strains isolated from China. The *MTL* loci (*MTL***a** or *MTL*α) of the twenty *C. auris* strains isolated from China were determined by PCR chemotyping.

*C. auris* is predominantly a haploid fungus. PCR analyses indicate that only BJCA001 is an *MTL***a** strain, while the isolates from Xiamen (XM1803 and XM1805) and other Chinese hospitals are *MTL*α strains (Figure 2). Interestingly, only strain BJCA001 was susceptible to all antifungal drugs tested; the other nineteen strains exhibited resistance to one antifungal drug (fluconazole) or additional antifungal drugs based on their MIC breakpoints (Table 1). Previous studies in *C. auris* indicate that there is an association between fluconazole resistance and the presence of hotspot mutations in the Erg11 lanosterol demethylase (e.g. Y132F, K143R, and VF125AL) (Lockhart et al. 2017; Du et al. 2020). Consistently, no hotspot mutations were found in strain BJCA001, while a hotspot mutation in Erg11 (VF125AL) was found in all of the other nineteen strains from China (Table 1).

**Table 1.** MICs and Erg11 mutations of the 20 *C.* *auris* clinical isolates from China

MIC (μg/mL)	FLC	ITC	POS	VRC	AMB	5-FC	AFG	CAS	MFG	Data adapted from	Erg11 mutation hot spot
XM1803	128	0.5	0.125	0.5	2	0.125	0.5	0.25	0.125	This study	VF125AL
XM1805	128	0.5	0.25	0.5	2	0.125	0.5	0.5	0.25	This study	VF125AL
BJCA001	2	0.03	0.02	0.0	0.25	<0.06	0.12	0.06	0.06	Reference 1	No mutation
C1921	128	0.25	0.25	0.5	2	0.125	0.5	0.5	0.25	This study	VF125AL
C1922	128	0.5	0.25	0.5	2	0.125	1	0.5	0.25	This study	VF125AL
RICU1	256	0.06	0.03	0.5	0.5	<0.06	0.03	0.06	0.06	Reference 2	VF125AL
RICU2	256	0.12	0.06	1.0	0.5	<0.06	0.12	0.12	0.12	Reference 2	VF125AL
RICU3	256	0.06	0.03	0.5	0.5	<0.06	0.12	0.06	0.06	Reference 2	VF125AL
RICU4	256	0.12	0.06	1.0	0.5	<0.06	0.12	0.12	0.12	Reference 2	VF125AL
RICU5	256	0.06	0.03	0.5	0.5	<0.06	0.12	0.12	0.12	Reference 2	VF125AL
RICU6	128	0.12	0.03	0.5	0.5	<0.06	0.12	0.12	0.12	Reference 2	VF125AL
RICU7	256	0.12	0.06	1.0	0.5	<0.06	0.12	0.12	0.12	Reference 2	VF125AL
RICU8	256	0.06	0.03	0.5	0.5	<0.06	0.12	0.06	0.06	Reference 2	VF125AL
NICU1	256	0.06	0.03	0.5	0.5	<0.06	0.12	0.06	0.06	Reference 2	VF125AL
NICU2	256	0.12	0.06	1.0	1	<0.06	0.12	0.12	0.12	Reference 2	VF125AL
NICU3	256	0.12	0.06	0.5	0.5	<0.06	0.12	0.12	0.12	Reference 2	VF125AL
NICU4	256	0.12	0.06	1.0	0.5	<0.06	0.12	0.12	0.12	Reference 2	VF125AL
NICU5	256	0.06	0.03	0.5	0.5	<0.06	0.12	0.06	0.06	Reference 2	VF125AL
NICU6	256	0.06	0.03	0.5	0.5	<0.06	0.12	0.06	0.06	Reference 2	VF125AL
NSICU1	256	0.06	0.03	0.5	0.5	<0.06	0.12	0.06	0.06	Reference 2	VF125AL

FLC fluconazole, ITC itraconazole, POS posaconazole, VRC voriconazole, AMB amphotericin B, 5-FC 5-flucytosine, AFG anidulafungin, CAS caspofungin, MFG micafungin, MIC minimal inhibitory concentration (μg/mL).

**References**

Wang XJ, Bing J, Zheng QS, Zhang FF, Liu JB, Yue HZ, Tao L, Du H, Wang YN, Wang H, et al. 2018. The first isolate of Candida auris in China: clinical and biological aspects. Emerg Microbes Infec. 7(1): 93. Tian S, Rong C, Nian H, Li F, Chu Y, Cheng S, Shang H. 2018. First cases and risk factors of super yeast Candida auris infection or colonisation from Shenyang, China. Emerg Microbes Infect. 7(1): 128.

**Table 2.** Clinical information of the 20 *C.* *auris* clinical isolates from mainland China

Location	Isolates name		Source	Year of Identification	Age/Gender	Reference
Xiamen	XM1803	Clade III	Drainage tube tip	2018	67‐year‐old, male	This study
	XM1805	Clade III	Blood	2018	67‐year‐old, male	This study
Beijing	BJCA001	Clade I	BALF	2018	76-year-old, female	1
	C1921	Clade III	Blood	2018	preterm male infants	2
	C1922	Clade III	Blood	2018	Preterm male infants	2
Shenyang	RICU1	Clade III	Urine	2017	70-year-old, male	3
	RICU2	Clade III	Urine	2017	69-year-old, female	3
	RICU3	Clade III	Sputum	2017	69-year-old, female	3
	RICU4	Clade III	Blood	2017	56-year-old, male	3
	RICU5	Clade III	Catheter	2017	82-year-old, female	3
	RICU6	Clade III	Catheter	2017	70-year-old, male	3
	RICU7	Clade III	Urine	2017	63-year-old, female	3
	RICU8	Clade III	Urine	2017	73-year-old, female	3
	NICU1	Clade III	Urine	2017	60-year-old, female	3
	NICU2	Clade III	Urine	2017	58-year-old, male	3
	NICU3	Clade III	Urine	2017	86-year-old, male	3
	NICU4	Clade III	Urine	2017	49-year-old, male	3
	NICU5	Clade III	Urine	2017	86-year-old, female	3
	NICU6	Clade III	Drainage	2017	82-year-old, female	3
	NSICU1	Clade III	Urine	2017	53-year-old, male	3
BALF, bronchoalveolar lavage fluid						

**Reference**

Wang XJ, Bing J, Zheng QS, Zhang FF, Liu JB, Yue HZ, Tao L, Du H, Wang YN, Wang H, et al. 2018. The first isolate of Candida auris in China: clinical and biological aspects. Emerg Microbes Infec. 7(1): 93. Chen Y, Zhao JY, Han L, Qi LH, Fan WH, Liu J, Wang ZG, Xia X, Chen J, Zhang LL. 2018. Emergency of fungemia cases caused by fluconazole-resistant Candida auris in Beijing, China. Journal of Infection. 77(6): 569–571. Tian S, Rong C, Nian H, Li F, Chu Y, Cheng S, Shang H. 2018. First cases and risk factors of super yeast Candida auris infection or colonisation from Shenyang, China. Emerg Microbes Infect. 7(1): 128.

### 3.3. Morphological and Sap expression analyses

**Figure 3.**
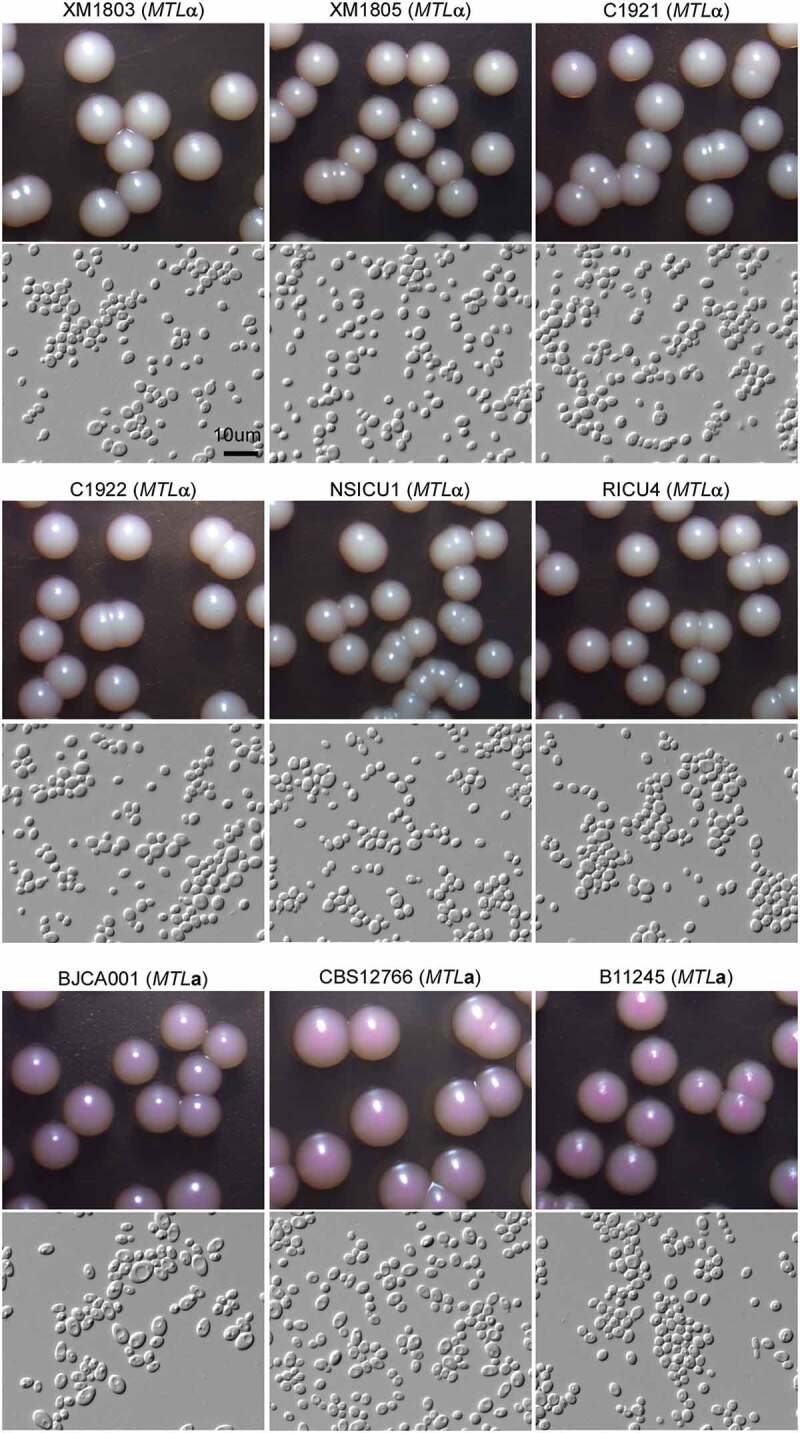
Colony and cellular morphologies of the different *C. auris* strains. Strains XM1803, XM1805, C1921, C1922, NSICU1, RICU4, and BJCA001 were isolated from China. The *MTL***a** strains, CBS12766 and B11245, were isolated from India and Venezuela, respectively. Yeast-form cells were plated onto YPD medium supplemented 5 μg/mL of phloxine B and incubated at 25°C for seven days. Scale bar = 10 μm.

We next performed morphological analyses on seven representative strains isolated from different hospitals in China (including six *MTL*α strains and one *MTL***a** strain). Two additional *MTL***a** strains (CBS12766 from India and B11245 from the United States (Chowdhary et al. 2013; Lockhart et al. 2017; Du et al. 2020)) were also included. As shown in Figure 3, the nine strains exhibited similar cellular morphologies when cultured at 30°C on YPD liquid medium containing the red dye phloxine B. These strains, however, differed in colony appearance on agar plates of the same medium. The colonies of the *MTL*α strains appeared white on YPD + phyloxine B plates, whereas those of the *MTL***a** strains appeared pink. These differences could be due to alterations in their cell wall structures or in their abilities to exclude the red dye. It remains to be investigated whether these differences are associated with antifungal resistance or virulence.

**Figure 4.**
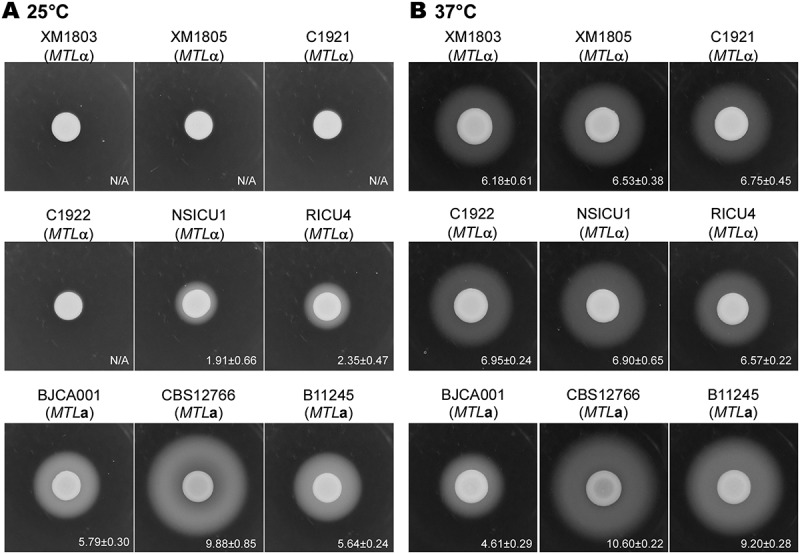
Sap expression for the different *C. auris* strains. Strains XM1803, XM1805, C1921, C1922, NSICU1, RICU4, and BJCA001 were isolated from China. The *MTL***a** strains, CBS12766 and B11245, were isolated from India and Venezuela, respectively. Approximately 5 × 10^6^ yeast-form cells of each strain in 5 μL ddH_2_O were spotted onto YCB-BSA medium plates and incubated at 25°C or 37°C for seven days. The white precipitation zones (halos) indicate the activity of the Saps. The widths (mm) of the zones were measured and shown below the corresponding image (n = 3). N/A = no obvious halos were observed.

To compare expression of the Sap virulence factors among the different *C. auris* isolates from China, we examined Sap expression levels using YCB-BSA assays (Wang et al. 2018). As shown in Figure 4, *MTL*α strains secreted a significantly lower level of Saps than *MTL***a** strains at 25°C. The expression of Saps in *MTL*α strains was significantly increased at 37°C, but it was still lower than that of CBS12766 and B11245 (*MTL***a** strains) at 37°C. Interestingly, strain BJCA001 exhibited a comparatively lower level of Sap expression relative to the other strains at 37°C. These results imply that the expression of Saps in *C. auris* could be associated with the mating type and regulated by environmental temperatures.

In the current study, we report a case of a *C. auris* candidemia in a patient in Xiamen, China. This represents the first emergence of this fungal pathogen in south China. The patient had a travel history to Japan; however, it is unlikely that the Xiamen strain originated from Japan given that Japan has only reported the presence of *C. auris* isolates of the East Asian clade. The Xiamen strain is closely related to the Shenyang and Beijing isolates of the South African clade; however, the patient had no travel history to these two cities. Therefore, the source of this infection remains to be determined.

We also compared the genetic and biological characteristics of existing *C. auris* strains isolated from China and found that there are several distinct features among the different strains, including the expression of virulence factors, antifungal susceptibilities, and cellular morphologies. Our findings indicate that the majority of clinical isolates (19/20, 95%) belong to the South African clade and are *MTL*α in mating type, whereas a single strain is associated with the South Asian clade and is *MTL***a** in mating type. Recently, additional *C. auris MTL*α isolates have been reported in Shenyang, China (Tian et al. 2021), while *MTL***a** isolates have been reported in Hong Kong, China (Tse et al. 2021). The Erg11 hotspot mutation (VF125AL) was found in all nineteen isolates of the South African clade, but not in the isolate of the South Asian clade (BJCA001). Interestingly, these clinical isolates all exhibited distinct cellular morphologies and differences in the levels of secretion of Saps. Taken together, there are two reported genetic clades of *C. auris* present in China and the different clinical isolates exhibit diversity in both biological and genetic features.

## Data Availability

All data generated or analysed during this study are included in this published article.
